# Design and Demonstration of Hingeless Pneumatic Actuators Inspired by Plants

**DOI:** 10.3390/biomimetics9100597

**Published:** 2024-10-01

**Authors:** Xiangli Zeng, Yingzhe Wang, Keisuke Morishima

**Affiliations:** Department of Mechanical Engineering, Osaka University, Osaka 565-0871, Japan; zengxiangli@live.mech.eng.osaka-u.ac.jp (X.Z.); ywang@live.mech.eng.osaka-u.ac.jp (Y.W.)

**Keywords:** pneumatic actuator, hingeless, soft robot, robots inspired by plants, birds-of-paradise plant, waterwheel plant

## Abstract

Soft robots have often been proposed for medical applications, creating human-friendly machines, and dedicated subject operation, and the pneumatic actuator is a representative example of such a robot. Plants, with their hingeless architecture, can take advantage of morphology to achieve a predetermined deformation. To improve the modes of motion, two pneumatic actuators that mimic the principles of the plants (the birds-of-paradise plant and the waterwheel plant) were designed, simulated, and tested using physical models in this study. The most common deformation pattern of the pneumatic actuator, bending deformation, was utilized and hingeless structures based on the plants were fabricated for a more complex motion of the lobes. Here, an ABP (actuator inspired by the birds-of-paradise plant) could bend its midrib downward to open the lobes, but an AWP (actuator inspired by the waterwheel plant) could bend its midrib upward to open the two lobes. In both the computational and physical models, the associated movements of the midrib and lobes could be observed and measured. As it lacks multiple parts that have to be assembled using joints, the actuator would be simpler to fabricate, have a variety of deformation modes, and be applicable in more fields.

## 1. Introduction

Plants are conventionally considered to grow slowly and live passively. Due to the imperceptibility of the changes they undergo, it is difficult to link plants with robots that require acute mobility. Thus far, animals and insects have inspired a number of robot designs, including legged [1,2,3], crawling [4,5,6], flapping [7,8,9], and swimming robots [10,11,12]. For such robots, controlling units, sensors, and actuators are necessary, considering the mechanism of how their prototype perceives and responds to stimulation. Most current robots have been fabricated with a rigid material to ensure resolution and robustness, which leads to environmentally unfriendly interactions that make them unsuitable for delicate subject operations, human care, and medical applications. Soft robots were proposed to solve this problem [13,14,15,16]. Two excellent examples of soft robots are pneumatic actuators that mimic the motion of octopuses [17,18] and that of fish [19,20], utilizing soft material and pressurized air. Nevertheless, the most common deformation pattern in pneumatic actuators is bending behavior. To improve their deformation modes, plants were selected as prototypes in this study. Plants can respond to environmental factors such as light [21], humidity [22], temperature [23], sound [24], gravity [25], and mechanical stimulation [26]. As they lack brains and muscles, plants move under the rules determined by the cellular structure [27]. Hingeless plants would enable more complex motion through joint-free design.

**Figure 1:**
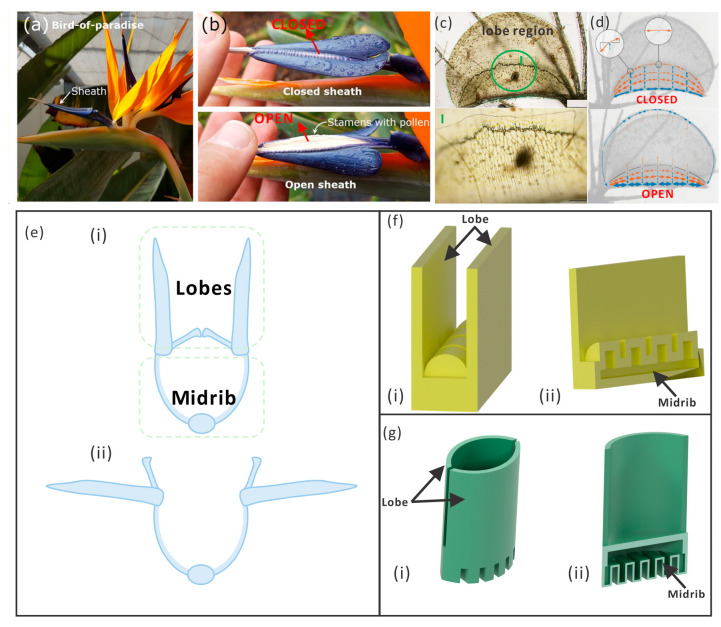
Different states of the bird of paradise and the waterwheel plant and the design of the actuators. (**a**) The sheath of the birds-of-paradise plant [34]. (**b**) The bending of the midrib causes the opening of the sheath lobes and exposes the stamens with pollen. (**c**) The optical image of the waterwheel plant. The lobe region has a concave shape when it is open and the motor region (I) has a convex shape. (**d**) When the lobes open, the midrib (motor region) bends downwards. (**e**) The cross-section view of the plants: (i) closed state; (ii) opening state. The cross-section views of the bird of paradise and the waterwheel plant are similar, but the midrib of the waterwheel plant has a convex shape. (**f**) The 3D CAD model of the ABP (actuator inspired by the birds-of-paradise plant): (i) 3D model; (ii) the half-section view of the ABP, where the thickness of the chamber wall was 3 mm. When the chamber was pressurized, the midrib would bend downwards. (**g**) The 3D CAD model of the AWP (actuator inspired by the waterwheel plant): (i) 3D model; (ii) the half-section view of the AWP, where the thickness of the chamber wall was 3 mm. When the chamber was pressurized, the midrib would bend upwards.

As shown in Figure 1, birds-of-paradise plants (Strelitzia reginae) open their lobes when a bird rests on their midrib based on their cellular construction, as observed by Eva et al. [28]. Lienhard et al. discovered that the opening of the lobes was directly related to the bending of the midrib, and this inspired the design of the architecture device Flectofin [29,30]. Similar phenomena occur on the waterwheel plant (Aldrovanda vesiculosa). Anna S. et al. investigated the deformation of the midrib and how the tension stored in the midrib is released when the lobes close [31,32,33]. The opening and closing of the lobes in both of these plants is induced through easily achieved bending, which could inspire the development of hingeless pneumatic actuators with more diverse functions. Without joint conjunctions as found in humans and animals, hingeless actuators could not only be less prone to wear and tear but also be imparted with more complex functions using artful structures and power sources. Additionally, it is possible to perform the one-step fabrication of a pneumatic actuator printed with additive manufacturing, which could reduce assembly costs and tolerances.

The cross-sections of these two plants are similar, as shown in Figure 1e. However, the bird of paradise has a straight midrib, while the midrib of the waterwheel plant has a convex shape. To obtain inspiration from plants for robot design, a simplified kinetic model of the plant was established, and then, following a similar principle, the mechanism of the two plants was explored in this study. The basic mechanism is the hybrid motion of the midrib and lobes, for which similar 3D models of two actuators are shown in Figure 1f,g. The midrib part of the actuator was pressurized and could bend; this bending deformation was transported to the lobes, causing them to open. Here, computational models were constructed to demonstrate the motion, deformation details, and stress distribution. The physical model was fabricated through 3D printing, in which silicon was used for the main body and paraffin was used as the sacrificed material. The behavior of the physical and computational models could be compared.

## 2. Mechanism and Fabrication Method

To mimic the principle of motion of the birds-of-paradise plant and the waterwheel plant, two kinds of pneumatic actuators were designed, simulated, and fabricated. Bending deformation could be easily achieved with pressurized asymmetric cushions; therefore, the midrib of the actuator was designed to bend with pressure. First, the CAD model was constructed using Inventor (Autodesk Inventor 2025). Then, the computational model was examined in COMSOL (COMSOL Multiphysics 6.1), in which a test cube was fabricated for parameter calibration. Lastly, the physical model was reversed by the 3D printing negative model. 

### 2.1. Design of the Pneumatic Actuators Inspired by Plants

**Figure 2:**
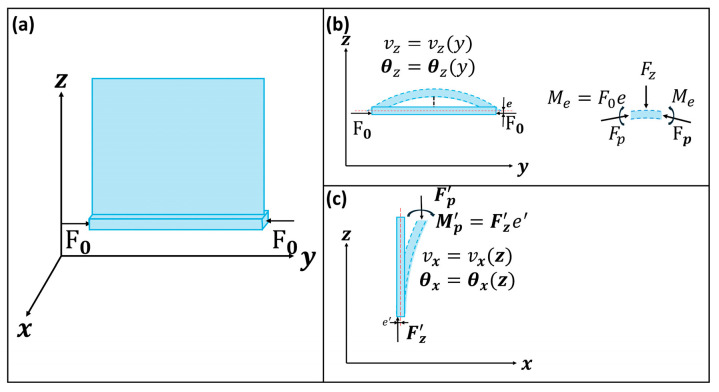
The kinetic model of the actuators. (**a**) The model is composed of a lobe fixed on a beam and the beam will bend under the $F_{0}$. (**b**,**c**) show the internal force condition of the model.

As shown in Figure 2, let us consider a model composed of a beam and a lobe fixed on the beam, where the beam bends under the external force $F_{0}$. Due to the asymmetric structure of the model, as shown in Figure 1d,e, there is an eccentric distance e, and the bending and buckling might happen at the same time in the model. First, the deformation of the beam $v_{z}(y)$ and rotation angle $\theta_{z}(y)$ can be calculated before buckling of the beam.

The internal force is:

(1) $$F_{p}\left(y\right)=\frac{F_{0}}{cos\theta_{z}(y)}\;$$

The torque $M_{p}$ is:

(2) $$M_{p}=F_{0}e+F_{p}\left(y\right)v_{z}=F_{0}e+\frac{F_{0}v_{z}\left(y\right)}{cos\theta_{z}(y)}$$

According to the equation:

(3) $$M_{p}=-EI\frac{d^{2}v_{z}\left(y\right)}{dy^{2}}$$

where E is the Young’s modulus of the material and I is the moment of inertia of the beam. When the deformation is small, Equation (3) can be expressed as follows:

(4) $$\frac{d^{2}v_{z}\left(y\right)}{dy^{2}}+k^{2}v_{z}\left(y\right)=-k^{2}e$$

in which $k^{2}=\frac{F_{0}}{EI}$. For Equation (4), the solution should be the general solution of the homogeneous equation plus a particular solution of the inhomogeneous part.

Finally, based on the boundary condition, the equation could be solved as:

(5) $$v_{z}\left(y\right)=e\left(tan(\frac{kl}{2})sin(ky)+cos(ky)-1\right)$$

where l is the length of the beam.

Then, the inner stress caused by the lobe can be calculated.

(6) $$F_{z}={2F}_{p}\left(y\right)sin\theta_{z}\left(y\right)=2F_{0}e\left(tan(\frac{k^{2}l}{2})cos(ky)-ksin(ky)\right)$$

$F_{p}$ causes the internal force $F_{z}^{\prime }$. Because the lobe is not in the center of the beam, there is also an eccentric distance, and when the lobe deviates from the balance position, the torque formed by $F_{p}^{\prime }$ and $M_{p}^{\prime }$ makes the lobe bend more. 

According to the same calculation as for the beam, the deformation of the lobe can be deduced.

(7) $$v_{x}\left(z\right)=e^{\prime }\left(tan(\frac{k^{\prime }l^{\prime }}{2})sin(k^{\prime }z)+cos(k^{\prime }z)-1\right)$$

where $k^{\prime }=\frac{F_{z}}{EI^{\prime }}$. $I^{\prime }$ means the moment of inertia of the lobe and $l^{\prime }$ means the length of the lobe. However, once $F_{0}$ is large enough to cause the buckling of the model, the whole system loses its balance.

According to the mechanism mentioned above, both actuators mimic the birds-of-paradise plant (ABP) and the actuator inspired by the waterwheel plant (AWP) can open its lobes; however, they differ greatly in that the AWP exhibits concave-shaped lobes, compared to the zero curvature of the ABP’s lobes. This causes an opposite deformation pattern where the ABP opens lobes once the midrib bends upwards, and the AWP opens when its midrib bends downwards. The structure is presented in detail in Figure 1c,f. The dimensions of the ABP and AWP were 100 mm×65 mm×30 mm and 67 mm×45 mm×40 mm, respectively, while the thickness of the wall was 3 mm for the pneumatic chambers.

### 2.2. Simulation Model of the Actuators

**Figure 3:**
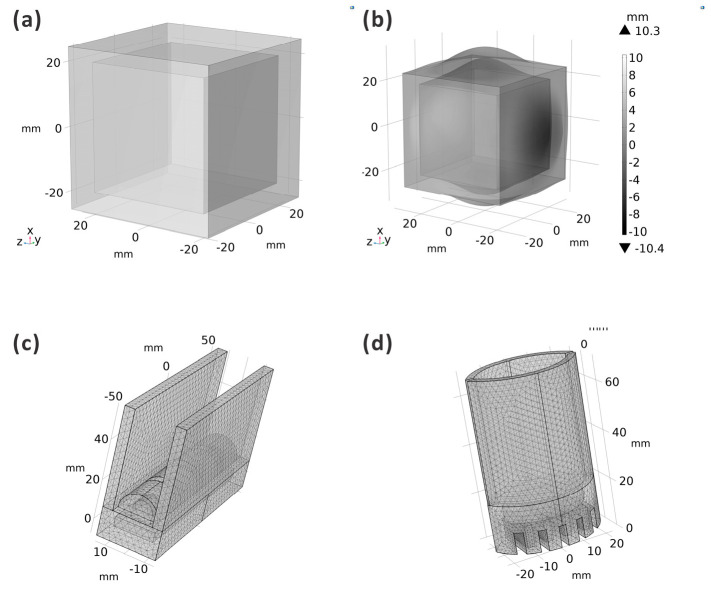
The computational models of the actuators. (**a**) The test cube for the material parameters. (**b**) The deformation results of the test cube. The material parameters were adjusted until the simulation results matched those of the physical model. (**c**,**d**) show the meshed computational model of the ABP (actuator inspired by the birds-of-paradise plant) and the AWP (actuator inspired by the waterwheel plant).

First, to calibrate the parameters of the material, a cube with a chamber was simulated and fabricated. The deformation was compared to ensure the precision of the computational model. As shown in Figure 3a, the cube’s dimensions were 50 mm×50 mm×50 mm and those of the inner chamber were 40 mm×40 mm×40 mm. The deformation result is shown in Figure 3b and indicates that the material’s parameters were $E=8\times 10^{5}\;Pa,\mu =0.48,and\;\rho =10^{3}\;kg/m^{3}$. All the computational models adopted these parameter values. The meshed computational models of the ABP and AWP are shown in Figure 2c,d, respectively. Both actuators were fixed at the central line of the midrib to ensure symmetrical motion.

### 2.3. Fabrication of the Actuators

**Figure 4:**
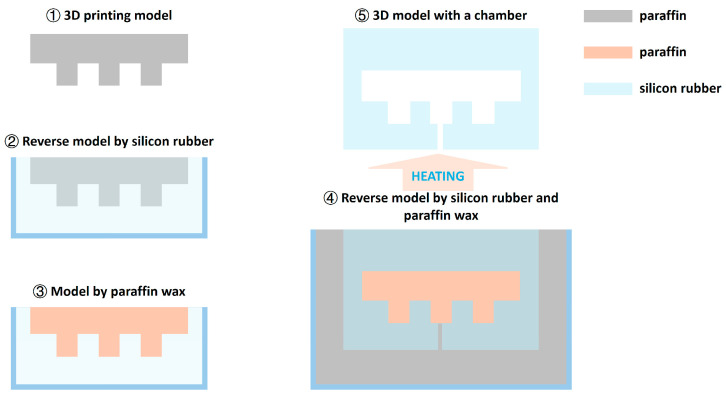
The fabrication method for the pneumatic actuators. The model was printed with polylactic acid (PLA) material, paraffin was used as the sacrificed material which could be melted after heating, and the main structure of the actuator was reversed using silicon.

As shown in Figure 4, the physical models with a chamber were fabricated using silicon, and paraffin was used as the sacrificed material. In Step 1, the same model for the chamber shape was prepared using 3D printing (material extrusion-based 3D printing with Flashforge Creator 3 Pro; material: polylactic acid). In Step 2, the model from the previous step was reversed using silicon. In Step 3, melted paraffin wax was poured into the model from the previous step. In Step 4, a 3D printing model designed for the final structure was reserved using the silicon and paraffin model from the previous step. In Step 5, this reversed model was heated to melt wax in the structure. Once the wax was melted, it outflowed from the structure.

The silicon contained liquids A and B (viscosity: A (6100 cps), B (5900 cps); density: 1.08 g/cm^3^; ingredients: vinyl silicone (62%), silicon 2,9,16,23-tellurium-tert-putiru 29H (33%), platinum (1%), and borane (methyl hydrogen siloxane) (4%)), which were mixed together when used, and this liquid solidified after several hours. In addition, liquid silicon could be shaped on the surface of the solid silicon, which meant that the top part could be fabricated after the bottom part.

## 3. Results

In the simulation model, the deformation of the models under different pressures was computed and compared. At the same time, the curve of the midrib could be examined carefully to inspect the coupling of the midrib and lobes. In the physical model, the motion of the pressurized model was recorded using a high-speed camera.

### 3.1. Simulation Results

#### 3.1.1. Simulation Results of the ABP

**Figure 5:**
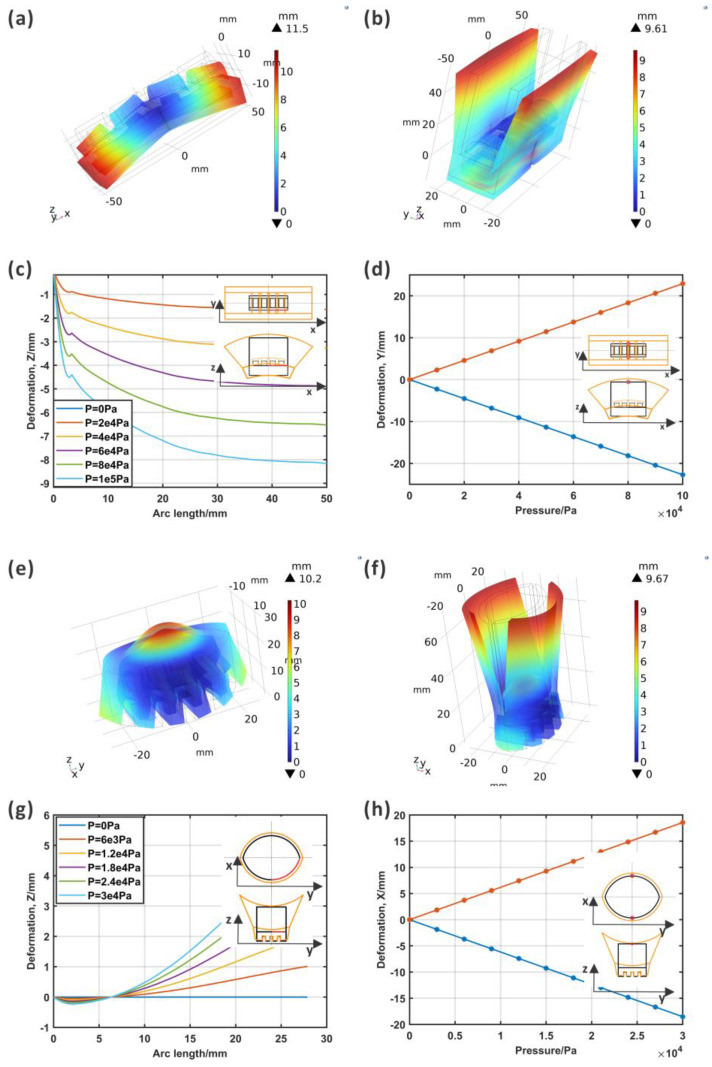
The computational model of the midrib of the ABP. (**a**) The deformation results for the midrib and two ends showed the largest deformation; (**b**) the midrib bent downward, and the lobes opened; (**c**) the midrib curve under different pressures, where the deformation increased as the pressure increased (the curve for P = 0Pa was coincident with the x-axis); (**d**) the opening of the lobes with the increased pressure. The computational model of the midrib of the AWP. (**e**) The deformation results for the midrib and two ends showed the largest deformation; (**f**) the midrib bent upward, and the lobes opened; (**g**) the midrib curve under different pressures, where the deformation increased as the pressure increased; (**h**) the opening of the lobes with the increased pressure.

In the simulation model, pressure could cause bending of the midrib, which should cause a coupling motion of the lobes. The bending of the midrib without lobes was calculated, and the results are shown in Figure 5a. The model without lobes was fixed from the central lines. The pressure could bend the midrib, the fixed part had the greatest stress, and the two ends had the greatest deformation. Meanwhile, the deformation increased with the pressure. 

After the simulation of the midrib, the model with lobes was computed (detailed results are shown in Movie S1: the computational results of ABP_stress and Movie S2: the computational results of ABP_deformation). The deformation in Figure 5b shows that the lobes opened symmetrically. Aiming to understand the relationship between the motion of the midrib and lobes, the deformation curve of the midrib and the distance of the two lobes are shown in detail in Figure 5c,d. In the line result for the midrib, it can be observed that not only did the deformation increase with the pressure, but there was also an inflection 3 mm from the central point. This was because of the counterpart of the upward deformation of the center of the actuator and the downward deformation of the two ends. In addition, the maximum deformation for the model with lobes was smaller than that of the model without lobes. Part of the deformation of the midrib was transmitted to the lobes. The distance between the two lobes also increased with the pressure, and the relationship presented as linear.

#### 3.1.2. Simulation Results of the AWP

Identically to the computational model of the ABP, the midrib was simulated first for the AWP. Figure 5e shows the 3D model and deformation information. The asymmetric structure helped in the bending.

For the simulation model with lobes, the bending of the midrib and the opening of the lobes took place simultaneously (detailed results are shown in Movie S3: the computational results of AWP_stress and Movie S4: the computational results of AWP_deformation). The deformations are presented in Figure 5f. Similar to that for the ABP, the line result for the midrib shows that the deformation increased with the pressure, but the bending displacement was smaller than that of the midrib model due to the integration of the lobes, as shown in Figure 5g. Figure 5g shows that there was a node with zero deformation where all the lines converged together, indicating that there must be a nodal line which would be fixed in the movement process. As shown in Figure 5h, the distance between the lobes increased with the pressure.

### 3.2. Physical Model Results

**Figure 6:**
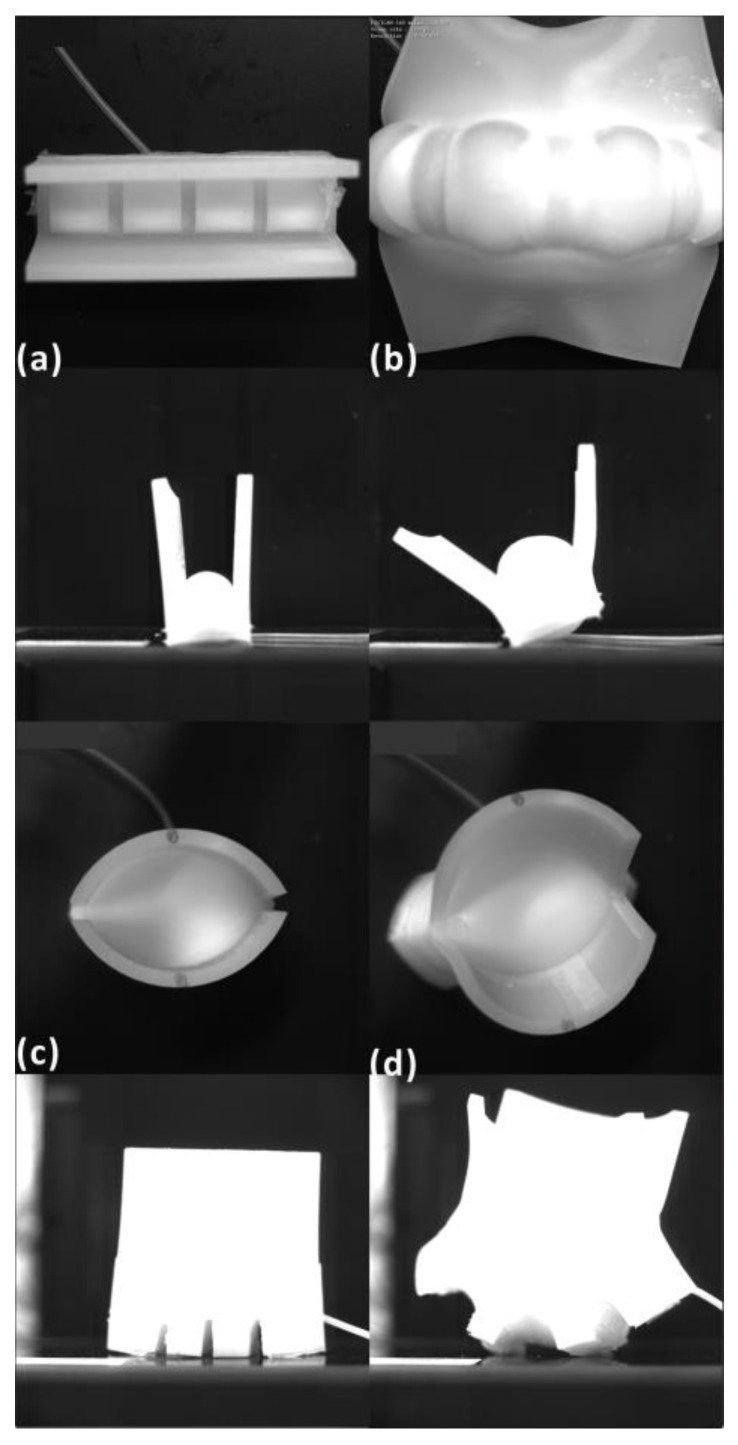
The physical models of the ABP and AWP: (**a**) pressure-free state of the ABP from the top and lateral views; (**b**) pressurized state of the ABP; (**c**) pressure-free state of the AWP from the top and lateral views; (**d**) pressurized state of the AWP.

The physical models were fabricated following the step shown in Figure 4, but the printed models were optimized for different actuators and sizes. The motion of the actuator was captured using a high-speed camera (Photron, SA3, PHOTRON LIMITED, Kyoto, Japan) and analyzed using motion software (Diip_Motion V 1.1.29). Figure 6 presents the prototype in unpressurized states and pressurized states. It can be seen that the actuators bent their midrib and opened their lobes under pressure. The tracking data were obtained to obtain more movement information.

#### 3.2.1. Results of the ABP

**Figure 7:**
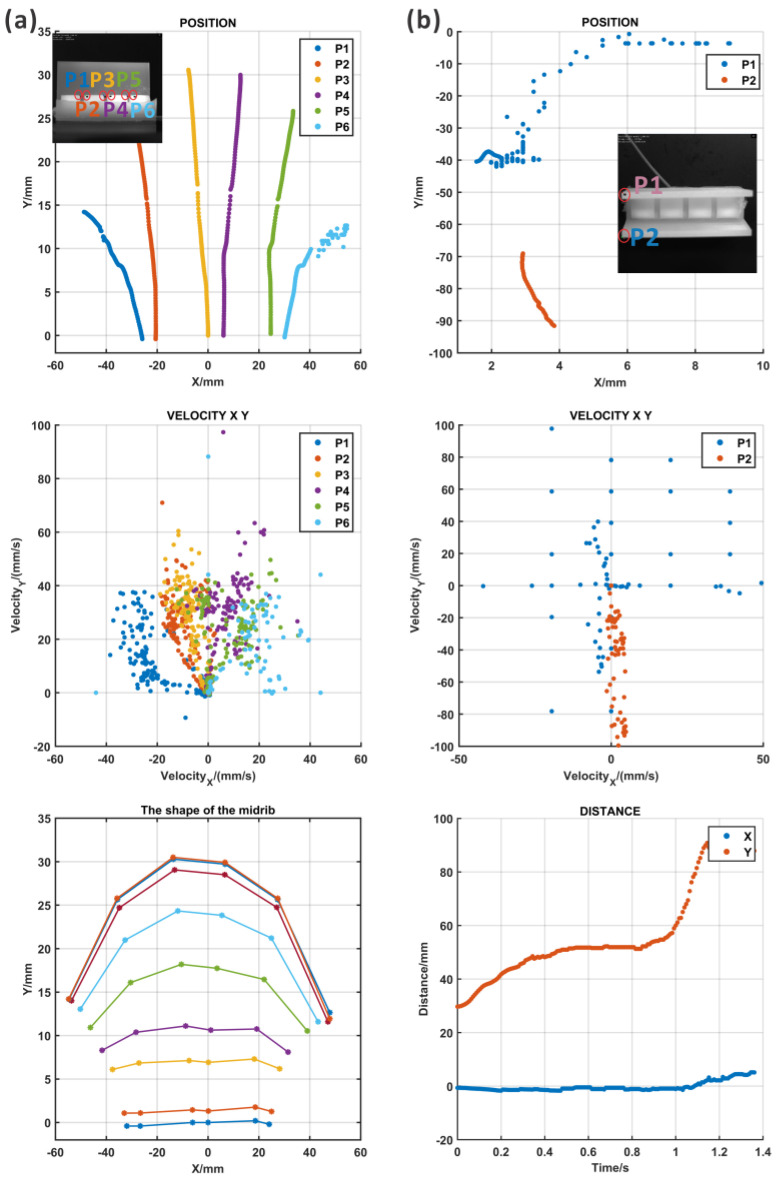
The motion tracking results for the ABP from the top as determined using a high-speed camera. (**a**) The first column: the marking points in the midrib and their movement paths, the composite velocity, and the shape of the midrib; (**b**) the second column: the marking points in the lobes and their movement paths, the composite velocity, and the distance between two lobes.

The motion of the ABP could be defined by the expansion of the midrib and the opening of the lobes from the top (Movie S5: the high-speed camera recording results of ABP_top). Two points from the lobes were tracked, and the lobe in which point 2 was located opened first; then, another lobe opened according to Figure 7b and the recording movie. In addition, the deformation took place almost in the Y direction and the distance between the two lobes increased in the motion process. The speed in the Y direction of the motion for the lobe with point 2 was faster in the first stage and slower later.

In the movie recording, it could be found that the actuator rotated in the pressurization process, which was caused by the asymmetry formed using the multi-step fabrication method. It was difficult to maintain the balance of the actuator under pressure. Meanwhile, the lobe in which point 1 was located snapped.

However, the bending of the midrib was difficult to image based on the current views; thus, one of the lobes was removed and the midrib was exposed for recording (Movie S6: the high-speed camera recording results for ABP_front without lobe). As shown in Figure 7a, six points were recorded for the deformation. There was some movement in both the X and Y directions, and the bending of the midrib could be examined using the curves composed of the tracked points, which is shown in the right figure of Figure 7a. In the results of the midrib curves, the midrib was flat at first, and then, with an increase in the pressure, the curvature increased. Although the motions of the midrib and the lobes were captured in two different movement processes, the bending of the midrib and the opening of the lobes clearly took place simultaneously.

#### 3.2.2. Results of the AWP

**Figure 8:**
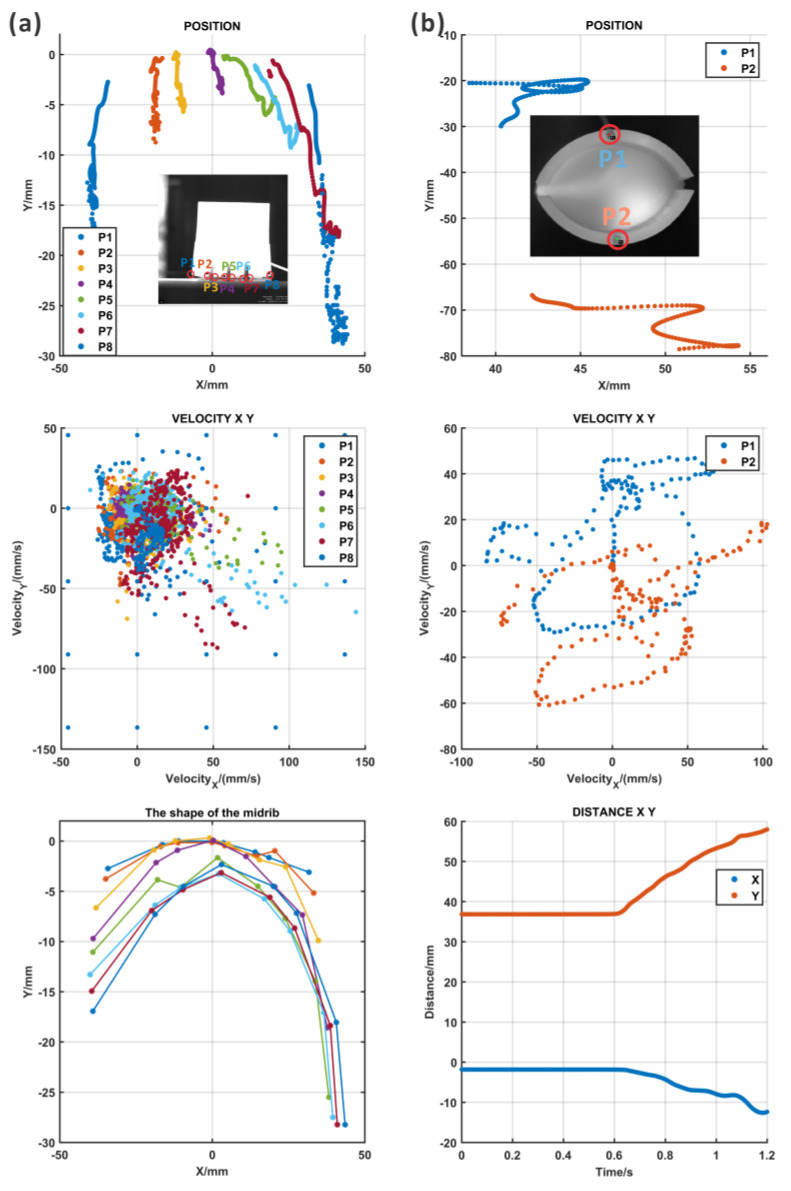
The motion tracking results for the AWP as determined from a top view with a high-speed camera. (**a**) The first column: the marking points in the midrib and their movement paths, the composite velocity, and the shape of the midrib; (**b**) the second column: the marking points in the lobes and their movement paths, the composite velocity, and the distance between two lobes.

Two points from each lobe and three points from the midrib were marked and tracked (Movie S7: the high-speed camera recording results of AWP_top). The tracking paths are shown in Figure 8b, and the midrib expanded to open the lobes, which could be demonstrated by the large distance between two points on the lobe. The velocity information over time could be evaluated in the middle figure of Figure 8b. According to the results in Figure 8b, a large deformation in both the X and Y directions appeared at point 2, but point 1 had a smaller X deformation. The reason for the asymmetric motion of points 1 and 2 was that the whole actuator rotated on the table during the motion process due to the expansion of the midrib. In addition, the fabrication process generated many errors such as differing thicknesses of the silicon because of the several fabrication steps, and the difficulty in ensuring perfect symmetry during assembly. This kind of fabrication problem could be solved through soft material 3D printing, which was costly but precise.

At the same time, the lateral view of the pressurization of the AWP was recorded to identify the bending of the midrib (Movie S8: the high-speed camera recording results of AWP_lateral). As imaged in the movie, the midrib bent, and the curvature increased during motion. Here, eight points were tracked during movement and their position, speed, and acceleration are analyzed in Figure 8a. The path indicates that all the points clearly moved along the X and Y directions, which represented the bending and expansion of the midrib. Similar to the top view, there was still some asymmetry in the motion caused by the rotation of the actuator. However, based on the fitting lines of eight tracked points, it can be seen that the degree of the bending of the midrib increased with the pressure.

## 4. Discussion

In the computational models, the transient analysis was performed in both actuators to compare their motion process with that of physical models. In the model of ABP, the actuator deformed when the pressure increased from 0 to 0.2 MPa within one second. The computational AWP deformed when the pressure increased to 0.03 MPa within one second. In the movies of the physical models, the actuators did not deform stably. ABP and AWP rotated during pressurization. The rotation was caused by the not completely symmetrical structure and unstable supporting method. The multi-step fabrication method led to a size error in the structure, and both actuators were pressurized and recorded on the surface of the table, which generated differences with the fixed method in the computational models.

**Figure 9:**
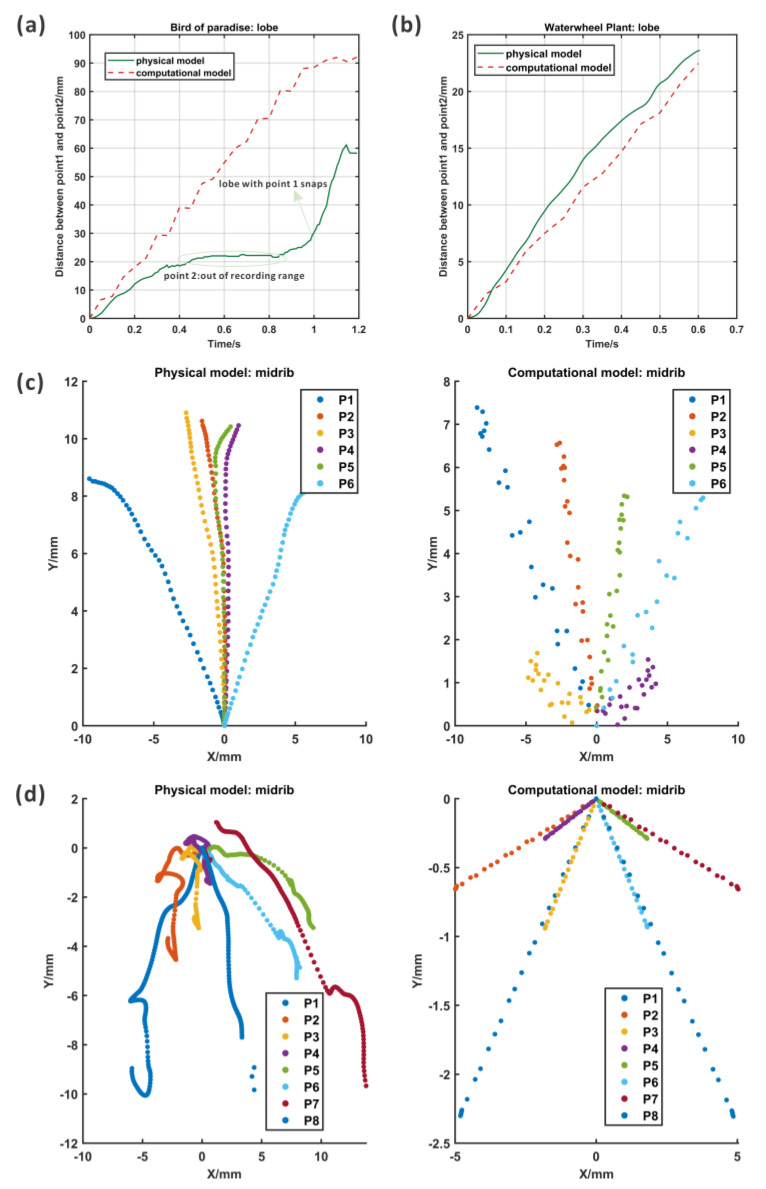
The comparison of the computational and physical results of ABP. (**a**) The first row, first column; (**c**) the second row: the deformation of the lobes and midrib, respectively, for the computational model and physical model. The comparison of the computational and physical results for ABP. (**b**) The first row, second column; (**d**) the third row: the deformation of the lobes and midrib, respectively, for the computational model and physical model.

For the ABP, as shown in Figure 9a, the deformation of two points on the lobes increased with pressure, which was coordinated with the trend that the distance between two lobes in the physical model increased. The computational model moved smoothly, but the physical model opened one lobe first and the other lobe snapped a little. The distance was almost kept the same from 0.5 s to 0.8 s, which was because the tracked point was out of the tracking range of the camera. In addition, the motion of the midrib was compared as shown in Figure 9c. The deformation of points from the midrib spread, even though the deformation of the physical model was greater than that of the computational model. Considering that the computational model of the lobes had greater deformation than the physical model, there might be some incomplete conversion.

Similarly, the motion of the AWP was also examined using computational and physical models, as shown in Figure 9b,d. Due to the rotation of the whole actuator, two points on the lobes showed large X direction movement in the physical model, but the computational model had no deformation in the X direction. However, the distance between two points in the physical model showed great consistency with the results of the computational model. The bending of the midrib had the same pattern as the midrib of the ABP, but some rotation occurred in the physical model.

**Figure 10:**
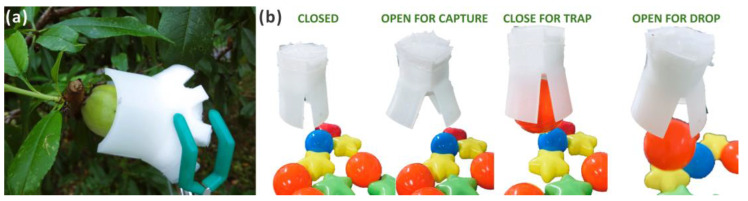
The demonstration of the actuator as a gripper. (**a**) Using AWP to harvest fruit. (**b**) Using AWP to trap and drop a light ball.

The computational and physical models shared the same deformation pattern, and the unstable motion of the physical models could be improved through one-step fabrication and a better fixation method. Ideally, the hingeless pneumatic actuator could act as a soft end for the rigid arm to achieve more functions such as fruit harvesting and light target manipulation (Movie S9: ball releasing), as shown in Figure 10. Moreover, taking advantage of the flexibility, biocompatibility, and adaptivity of the soft actuators proposed in this study, they show great potential for application in the field of surgery robots, human–machine interactions, and complex environment rescue.

Plants always take advantage of morphology to perform different functions such as phalaenopsis to deceive insects into pollinating them because of their puzzling appearance, similar to the female insect. As a hingeless species, all the organs of the plants grow continuously and their morphology is determined by cellular topology. Uncovering how the plants control their whole motion by integrative construction could enlighten hingeless mechanical design.

## 5. Conclusions

In this paper, two plants (the birds-of-paradise plant and the waterwheel plant) are discussed with regard to their movement principles. Even though they both utilize the bending of the midrib to actuate the opening of the lobes, the bending direction was found to be opposite in contribution to the morphology. Following the same principle, two actuators were designed, in which asymmetric structures with cushions were adopted to accomplish bending deformation. In order to understand the coupling motion between the midrib and lobes, the computational and physical models were tested and compared. In the simulation models, the downward bending in the ABP opened its lobes; however, the upward bending did the same thing in the AWP. This meant that a much more complex design may lead to diverse motion patterns. In the physical models, the motions of the ABP and AWP were captured from different views using a high-speed camera. The marked points could be tracked, and their movement paths and velocity were analyzed. The curvature of the midrib for both actuators increased when it bent, and the distance between the two lobes also increased. Human- and animal-inspired soft robotics often feature joints, which can concentrate stress in specific areas, resulting in faster wear and tear. In contrast, plant-inspired robotics move as a whole structure without joints, thereby reducing wear over time. The hingeless actuator proposed in this study presented a unique deformation pattern compared to previous pneumatic actuators, and the investigation of the movement principle of the plants could reveal more designs for soft robots.

## Data Availability

The data are contained within the article.

## Supplementary Materials

The following supporting information can be downloaded at: https://www.mdpi.com/article/10.3390/biomimetics9100597/s1, Movie S1: the computational results of ABP_stress; Movie S2: the computational results of ABP_deformation; Movie S3: the computational results of AWP_stress; Movie S4: the computational results of AWP_deformation; Movie S5: the high-speed camera recording results of ABP_top; Movie S6: the high-speed camera recording results of ABP_front; Movie S7: the high-speed camera recording results of AWP_top; Movie S8: the high-speed camera recording results of AWP_lateral; Movie S9: ball releasing.
